# Efficacy of Oroxylin A in ameliorating renal fibrosis with emphasis on Sirt1 activation and TGF-β/Smad3 pathway modulation

**DOI:** 10.3389/fphar.2024.1499012

**Published:** 2024-12-02

**Authors:** Guangzhuang Li, Sentao Xian, Xianchao Cheng, Yunhua Hou, Wenqing Jia, Yukui Ma

**Affiliations:** ^1^ School of Bioengineering, Qilu University of Technology (Shandong Academy of Sciences), Jinan, Shandong, China; ^2^ School of Chemistry and Chemical Engineering, Qilu Normal University, Jinan, China; ^3^ Tianjin Key Laboratory on Technologies Enabling Development of Clinical Therapeutics and Diagnostics (Theranostics), School of Pharmacy, Tianjin Medical University, Tianjin, China

**Keywords:** Oroxylin A, chronic kidney disease, renal fibrosis, Sirt1, molecular docking

## Abstract

**Introduction:**

Renal fibrosis poses a serious threat to human health. At present, there are few types of traditional Chinese medicine used to treat this disease, and Oroxylin A (OA), as a natural product with multiple biological activities, is expected to be used for the treatment of renal fibrosis.

**Methods:**

The tolerance of osteoarthritis and its impact on renal fibrosis were studied through ADMET, Lipinski’s filter, establishment of a unilateral ureteral obstruction (UUO) model, and molecular docking.

**Results:**

OA has good drug tolerance. Compared with the sham group, UUO mice that did not receive OA treatment showed severe tubular dilation and atrophy, extracellular matrix (ECM) deposition, and inflammatory cell infiltration in their kidneys, while OA-treated mice showed significant improvement in these symptoms. OA treatment remarkably restrained the accumulation of fibronectin and α-SMA. Moreover, OA treatment remarkably decreased the abnormal upregulation of inflammatory factors (IL-1β, IL-6, and TNF-α) in the obstructed kidney of UUO mice. Sirtuin1 (Sirt1) expression was markedly diminished in the kidneys of UUO mice and TGF-β1-induced HK-2 cells, whereas this reduction was largely reversed after OA treatment. The results support that OA exerts antifibrotic effects partly through the promotion of the activity of Sirt1. In *in vitro* results, OA treatment markedly inhibited the activation of Smad3 in UUO mice, thereby ameliorating renal fibrosis. OA could form hydrogen bonds with key the amino acid ASN226 in Sirt1, thereby activating Sirt1, which might also be the reason why OA could resist renal fibrosis.

**Discussion:**

Our study indicated that OA might exert anti-renal fibrosis effects through the activation of Sirt1 and the suppression of the TGF-β/Smad3 signaling pathway.

## 1 Introduction

Chronic kidney disease (CKD) poses a serious threat to health. The incidence rate is increasing each year, and the main feature is renal fibrosis (Ledo et al., 2024; Speer et al., 2022; Ceccotti et al., 2024; Ruiz-Ortega et al., 2020; Miguel et al., 2021). In fibrotic kidneys, interstitial fibroblasts are the principal effector cells that produce the extracellular matrix (ECM), and their activation is an important pathogenetic event of renal fibrosis. The kidney’s interstitial fibroblasts differentiate into myofibroblasts during obstruction, resulting in excessive ECM synthesis and renal fibrosis. Despite numerous attempts, the underlying mechanisms of renal fibrosis remain unclear. Therefore, it is necessary to identify and develop effective anti-renal fibrosis drugs to prevent the progression of CKD.

Oroxylin A (OA) is a natural product derived from *Scutellaria baicalensis* (Chen et al., 2024). Reports show that OA has anti-inflammatory, anti-tumor, and vascular protective effects (Wang et al., 2024; Liu et al., 2020; Bai et al., 2021). Previous studies have found that OA markedly mitigates carbon tetrachloride-triggered liver fibrosis by activating autophagy (Chen et al., 2018). OA also exerts anti-inflammatory effects in liver fibrosis (Zhang et al., 2018; Shen et al., 2020). Moreover, OA attenuated the pyroptosis of hepatocytes by blocking mitochondrial ROS to promote PGC-1α/Mfn2 signaling. Oxidative stress and inflammation are the key factors in renal fibrosis (Oyama et al., 2021), and it has been found that OA may have anti-renal fibrosis functions.

**FIGURE 1:**
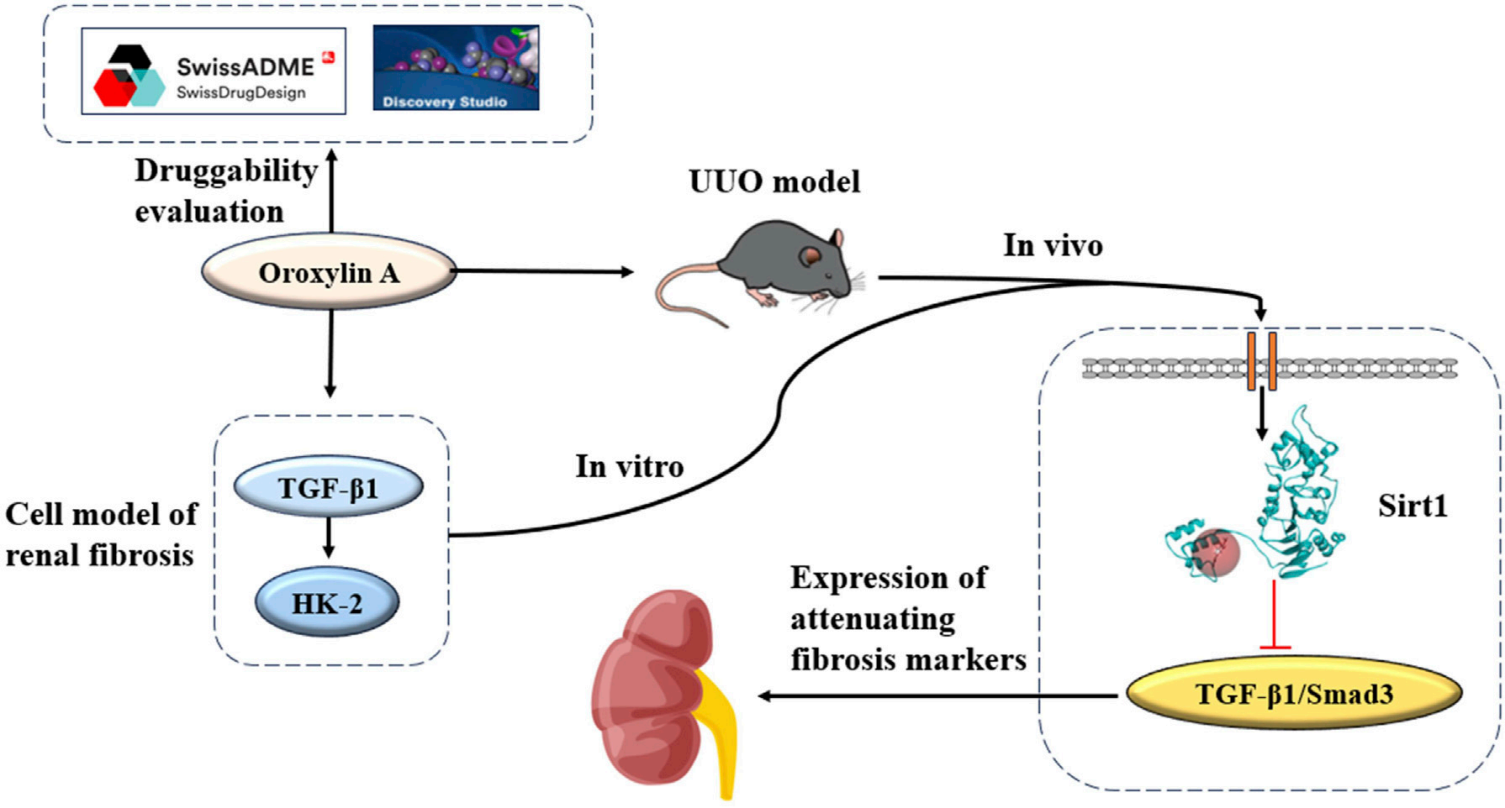
OA inhibits the TGF-β1/Smad3 signaling pathway by activating Sirt1.

Transforming growth factor-beta-1 (TGF-β1) emerges as a primary initiator of renal fibrosis. Once fibrogenesis is initiated, the secretion of TGF-β1 triggers the activation of receptors. The initiation of this process triggers a series of events, of which the most significant was the phosphorylation of Smad2/3, which leads to the transfer of activated p-Smad3 to the nucleus. This sequence of actions enhances the transcription of genes related to the ECM, thereby increasing matrix production (Sureshbabu et al., 2016). Based on these findings, therapeutic approaches targeting the TGF-β/Smad3 signaling pathway may provide a new avenue for treating CKD (Yu et al., 2022). Sirtuin1 (Sirt1) plays an important role in regulating the cell cycle, differentiation, apoptosis, and metabolism (Wang et al., 2023). Sirt1 is a key regulatory factor in various cellular processes, and it participates in the regulation of renal fibrosis. Studies indicate that activating Sirt1 may mitigate renal fibrosis, as evidenced by its capacity to attenuate fibrosis by repressing HIF-2α (Li et al., 2021). Conversely, in liver-specific contexts, the absence of Sirt1 exacerbates liver fibrosis, especially post-injury (Liu et al., 2017). Consequently, targeting Sirt1 through pharmacological modulators holds promise as a therapeutic approach for renal fibrosis. It was observed that OA exhibits renoprotective properties and possesses anti-renal fibrosis effects, primarily attributed to its activation of Sirt1. Conversely, the inhibition of Sirt1 may compromise the renoprotection of OA. We studied the durability of OA and the influence of OA on renal fibrosis, which mainly included ADMET, Lipinski’s filter, establishing a unilateral ureteral obstruction (UUO) model, and molecular docking (Figure 1).

## 2 Material and methods

### 2.1 ADMET prediction and Lipinski’s filter

The ADMET module in Discovery Studio 3.5 was used to predict the pharmacokinetics and toxicity of OA (Zhu et al., 2021). In ADMET prediction, the pharmacokinetics (water solubility, blood–brain barrier [BBB], cytochrome P450 2D6 [CYP2D6], liver toxicity, human intestinal absorption [HIA], and plasma protein binding [PPB]) of OA were studied (Ju et al., 2016). SwissADME (Daina et al., 2017) was used (http://www.swissadme.ch/index.php) to evaluate the physicochemical properties of OA molecular weight (MW), number of hydrogen bond acceptors (nHAs), number of hydrogen bond donors (nHBs), lipid water partition coefficient (ALogP), and number of rotatable bonds (nRot).

### 2.2 Animal model

The UUO model was established as described in a previous study (Zhong et al., 2024), and male C57BL/6 mice aged 10–12 weeks were selected. After anesthesia, the abdominal cavity was accessed through a midline incision, and double ligation was performed on the left ureter. The mice were randomly divided into three groups, with six animals in each group: sham operation (sham), where sham mice underwent ureteral exposure surgery without ligation of the ureter; UUO; and UUO + OA (Cat No. HY-N0560, purity = 99.74%, MedChemExpress, United States). After 14 days of induction with UUO, the mice were fasted overnight. The blood samples were collected from the eyeballs and centrifuged (3,000 r/min, 15 min), and serum was collected and stored. Then, the mice were euthanized by cervical dislocation. The animal experiments were approved by the Animal Ethics Committee (YSY-DWLL-2024438).

### 2.3 Cell culture

HK-2 cells were provided by Dr. Yong Liu (Chongqing University of Traditional Chinese Medicine, China). The HK-2 cells were cultured in DMEM/F12 medium (MeilunBio, China) and stored in a humidified chamber (37°C, 5%, CO_2_). To evaluate the anti-fibrotic properties of OA, OA was directly added to subcloned HK-2 cells and incubated. The HK-2 cells were subjected to a 24-h starvation period using DMEM containing 0.5% FBS and then exposed to TGF-β1 (10 ng/mL, PeproTech, United States) or Ex-527 (Shyuanye, China) for 24 h.

### 2.4 Real-time quantitative PCR

The RNAiso Plus reagent was used to extract RNA from kidneys and HK-2 cells. The cDNA was synthesized using the PrimeScriptTM RT Kit (Takara, Japan). A meausre of 2 μg RNA extracted from tissues and cells was used as a template for RT-PCR. RT-PCR was performed on cDNA using the SYBR Green method and QuantStudio. The amplification protocol was as follows: 30 s for the pre-read stage at 60°C, 10 min for denaturation at 95°C, 40 cycles of denaturation at 95°C for 15 s and annealing and extension at 60°C for 1 min, and then 30 s for post-read stage at 65°C. The Ct values for each sample were used in the analysis of 2^-△△Ct^ data. Table 1 lists the primers used.

**TABLE 1:** Primer sequences used in the study.

Gene	Forward	Reverse
m *Fibronectin*	CAG​CCA​GGC​ACT​GAC​TAC​AA	AGG​GGA​TCC​AGG​CTT​CTC​AT
m α-*SMA*	GTA​CCC​AGG​CAT​TGC​TGA​CA	GCT​GGA​AGG​TAG​ACA​GCG​AA
m *Il-1β*	TGC​CAC​CTT​TTG​ACA​GTG​ATG	AAG​GTC​CAC​GGG​AAA​GAC​AC
m *Il-6*	CAA​CGA​TGA​TGC​ACT​TGC​AGA	TGT​GAC​TCC​AGC​TTA​TCT​CTT​GG
m *TNF-*α	ATG​GCC​TCC​CTC​TCA​TCA​GT	TTG​CTA​CGA​CGT​GGG​CTA​C
m *Sirt1*	CGG​CTA​CCG​AGG​TCC​ATA​TAC	ACA​ATC​TGC​CAC​AGC​GTC​AT
m β-*Actin*	GTG​ACG​TTG​ACA​TCC​GTA​AAG​A	GCC​GGA​CTC​ATC​GTA​CTC​C
h *Sirt1*	CCT​ACT​GGC​CTG​AGG​TTG​A	GGA​CGG​AGG​AAA​AGA​GCG​AAT
h *ACTIN*	CAT​GTA​CGT​TGC​TAT​CCA​GGC	CTC​CTT​AAT​GTC​ACG​CAC​GAT

### 2.5 Histology staining

Conventional methods were used to prepare the paraffin-embedded sections of mouse kidneys. In brief, the kidneys were immersed in 4% paraformaldehyde solution for an extended period, followed by dehydration using a series of increasing ethanol concentrations. The kidneys were embedded in paraffin and cut into sections with a thickness of 4 μM. Then, these sections were dewaxed and stained to evaluate the histopathological changes in kidney tissue. The analysis was conducted using ImageJ software (Bethesda, Maryland, United States).

### 2.6 Immunofluorescence assay

The mouse kidney sections were incubated overnight at 4°C with fibronectin and α-SMA antibodies (Servicebio, China). Then, the kidney sections were stained with the Cy3 conjugated secondary antibody at 37°C for 1 h. DAPI was used to visualize the nuclei of cells. The slices were incubated with biotin-linked secondary antibodies at 37°C for 1 h. Antigen antibody reactions were visualized using a DAB substrate kit (ab64238, Abcam). The slides were counterstained with hematoxylin, dehydrated, and mounted.

### 2.7 Western blot analysis

Tissue and cell extracts were obtained using RIPA buffer. The BCA kit (Bio-Rad Lab, California, United States) was used to quantify the total protein concentration. The protein lysate was loaded onto 10% SDS-PAGE gel and transferred to polyvinylidene fluoride membranes (Bio-Rad Lab, California, United States). The membranes were then sealed with 5% skim milk for 1 h. Then, they were incubated with primary antibodies (Servicebio, China) anti-fibronectin (1:400), anti-α-SMA (1:1,000), anti-Sirt1 (1:1,000), anti-Smad3 (1:1,000), anti-p-Smad3 (1:1,000), and anti-β-Actin (1:1,000) at 4°C overnight. After being washed three times with TBST, the polyvinylidene fluoride membranes were exposed to a horseradish peroxidase-conjugated secondary antibody at a release degree of 1:2,000 (Servicebio, China) and incubated for 1 h at room temperature.

### 2.8 Statistical analyses

GraphPad Prism 9.0 (GraphPad Software, United States; http://www.graphpad.com/) was used to visualize and analyze data. The normality of the data was assessed using the Shapiro–Wilk test. One-way ANOVA was used to analyze the differences between groups, and Tukey’s *post hoc* test was used to determine the level of significant difference between each group in *post hoc* comparisons. All data are expressed as the mean ± sd. *p* < 0.05 was considered statistically significant (Yang et al., 2024).

### 2.9 Molecular docking

CDOCKER is a grid-based molecular docking method that uses a CHARMM force field (Jia et al., 2018). Sirt1 (ID: 4ZZH) was obtained from the PDB database (https://www.rcsb.org/). This study used Discovery Studio 3.5 software for molecular docking. Since the proteins downloaded from the PDB have missing residues, the “Prepare Proteins” module was used for protein repair, and the “From Current Selection” module was used to establish binding sites. Next, we prepared this protein, including dehydration, addition of hydrogen atoms, and addition of residue sequences. The binding site was a sphere with a radius of 9.27 Å and coordinates of X = 25.63, Y = −53.99, and Z = 2.23. SMILES of OA obtained from the PubChem database (https://pubchem.ncbi.nlm.nih.gov/) used the “Prepare Ligands” module to obtain the isomers of OA. The CDOCKER method was used to study the combination of OA and Sirt1. Finally, the results were visualized in the CDOCKER report and used for further analyses.

## 3 Results

### 3.1 ADMET prediction and Lipinski’s filter

**FIGURE 2:**
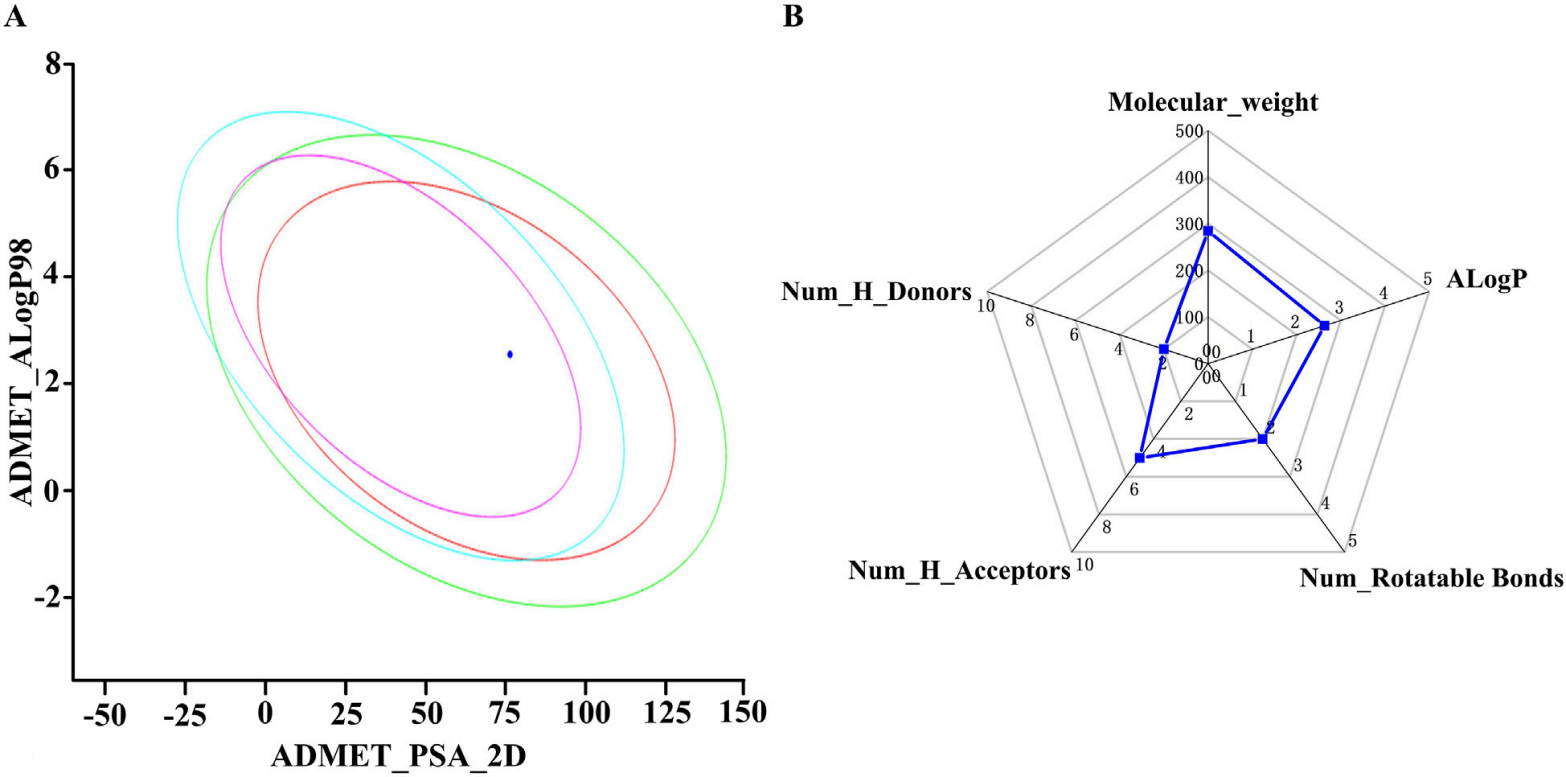
**(A)** Plot of polar surface area (PSA_2D) vs AlogP. **(B)** Analysis of Lipinski’s rule of five using a radar chart as a tool for investigated compounds.

The druggability of OA was researched. Figure 2A shows that OA had good HIA and BBB. The MW, nHA, nHD, nRot, and ALogP followed Lipinski’s rule (Figure 2B).

Next, ADMET characteristics of OA were evaluated (Table 2). Human intestinal absorption and solubility are two key factors that affect oral bioavailability. The solubility level of OA was 3, which indicated relatively good solubility. The absorption level of OA was 0, indicating high intestinal absorption. The inhibition of CYP2D6 by drugs constitutes the majority of cases of drug–drug interaction. It could be found that OA might not inhibit CYP2D6. In addition, the prediction results of toxicity (mouse female FDA, mouse male FDA, rat female FDA, rat male FDA, WOE prediction, Ames prediction, and skin irritancy) showed that OA had no risk of carcinogenicity, mutagenicity, and skin irritation (Table 3). So, the physicochemical and ADMET properties of OA were within acceptable ranges.

**TABLE 2:** ADMET prediction for OA.

ID number	Solubility level^a^	CYP450 2D6^b^	Absorption level^c^	PPB^d^
Oroxylin A	3	−2.79684	0	1.79429

^a^ Solubility_ level: 1, very low; 2, yes, low; 3, yes, good; and 4, optimal.

^b^ CYP450 2D6: <0.161, non-inhibitor; >0.161, inhibitor.

^c^ Absorption_ level: 0, good; 1, moderate; 2 low; and 3, very poor.

^d^ PPB: < −2.209, ≥90%; >-2.209, ≤90%.

**TABLE 3:** TOPKAT prediction for OA.

Mouse female FDA		Male mouse FDA		Rat female FDA		Rat male FDA		WOE prediction		Ames prediction		Skin irritancy
Probability	Effect	Probability	Effect	Probability	Effect	Probability	Effect	Probability	Effect	Probability	Effect	Effect
0.216984	NC	0.246201	NC	0.22312	NC	0.294397	NC	0.472273	NC	0.039966	NM	None

NC, non-carcinogen; NM, non-mutagen.

### 3.2 OA alleviated renal fibrosis progression by improving kidney function in UUO mice

**FIGURE 3:**
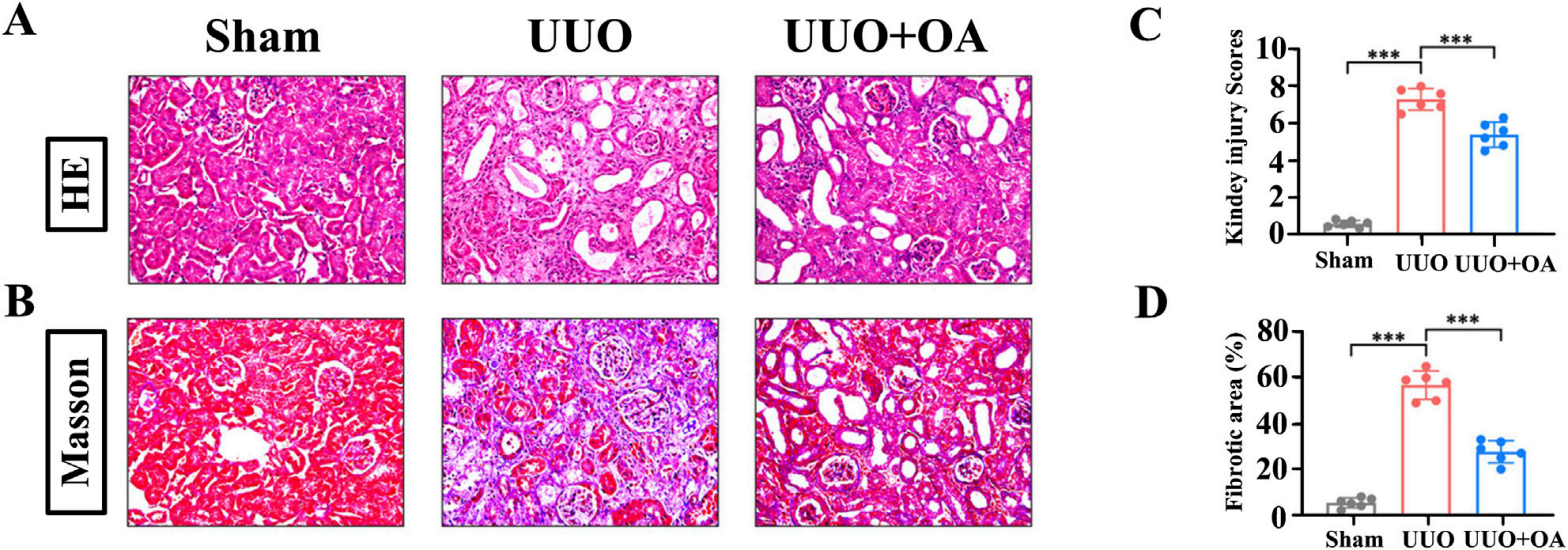
Oroxylin A ameliorates the deterioration of pathological damage in UUO mice. **(A)** Representative H&E staining. **(B)** Masson’s trichrome staining of UUO mice. The scale bar in the top panels corresponds to 200 μM. **(C, D)** Quantification of tubular injury and interstitial fibrosis area (n = 6). Data are represented as the mean ± sd. ***p < 0.001 and **p < 0.01.

**FIGURE 4:**
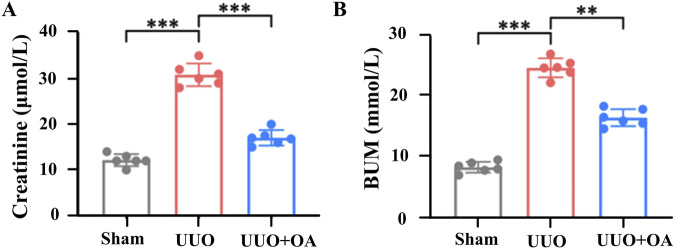
Oroxylin A ameliorates the deterioration of renal function in UUO mice. **(A, B)** Serum creatinine and blood urea nitrogen levels at 14 days after UUO surgery (n = 6). Data are represented as the mean ± sd. ***p < 0.001 and **p < 0.01.

UUO mice were orally administered OA (5 mg/kg/day, po), referring to previous studies (Kim et al., 2007). As shown in Figures 3A–D, HE and Masson’s staining showed that the obstructed kidneys in UUO mice without OA treatment displayed severe tubular dilation and atrophy, ECM deposition, and inflammatory cell infiltration. In contrast, these conditions were significantly ameliorated in OA-treated UUO mice. Additionally, OA treatment significantly reduced BUN and Scr levels in UUO mice, indicating that OA treatment improved renal function in UUO mice (Figures 4A, B). Altogether, these results indicated that OA can alleviate renal fibrosis in UUO mice.

### 3.3 OA treatment reduced the expression levels of fibronectin and α-SMA in UUO mice

**FIGURE 5:**
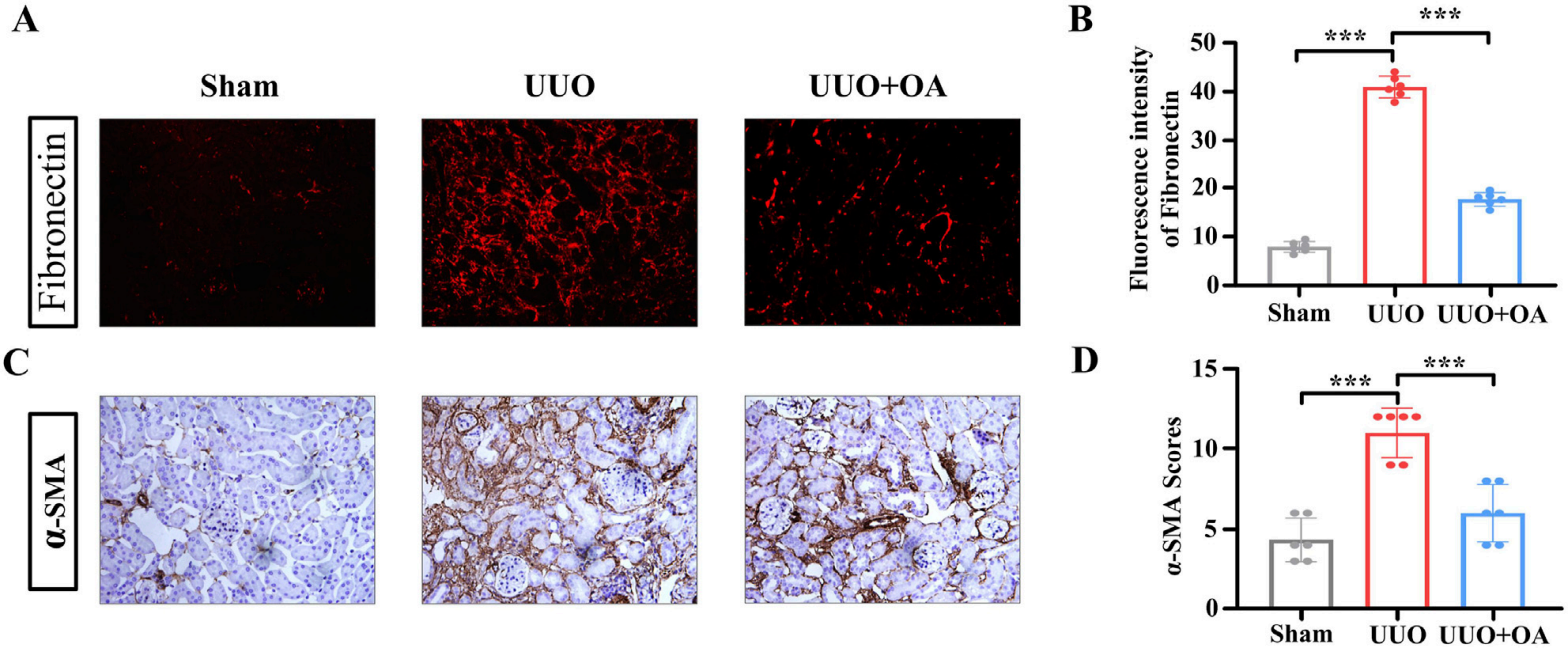
**(A–D)** Representative images of immunostaining for fibronectin and α-SMA in UUO mice (n = 6). Data are represented as the mean ± sd. ***p < 0.001 and **p < 0.01.

**FIGURE 6:**
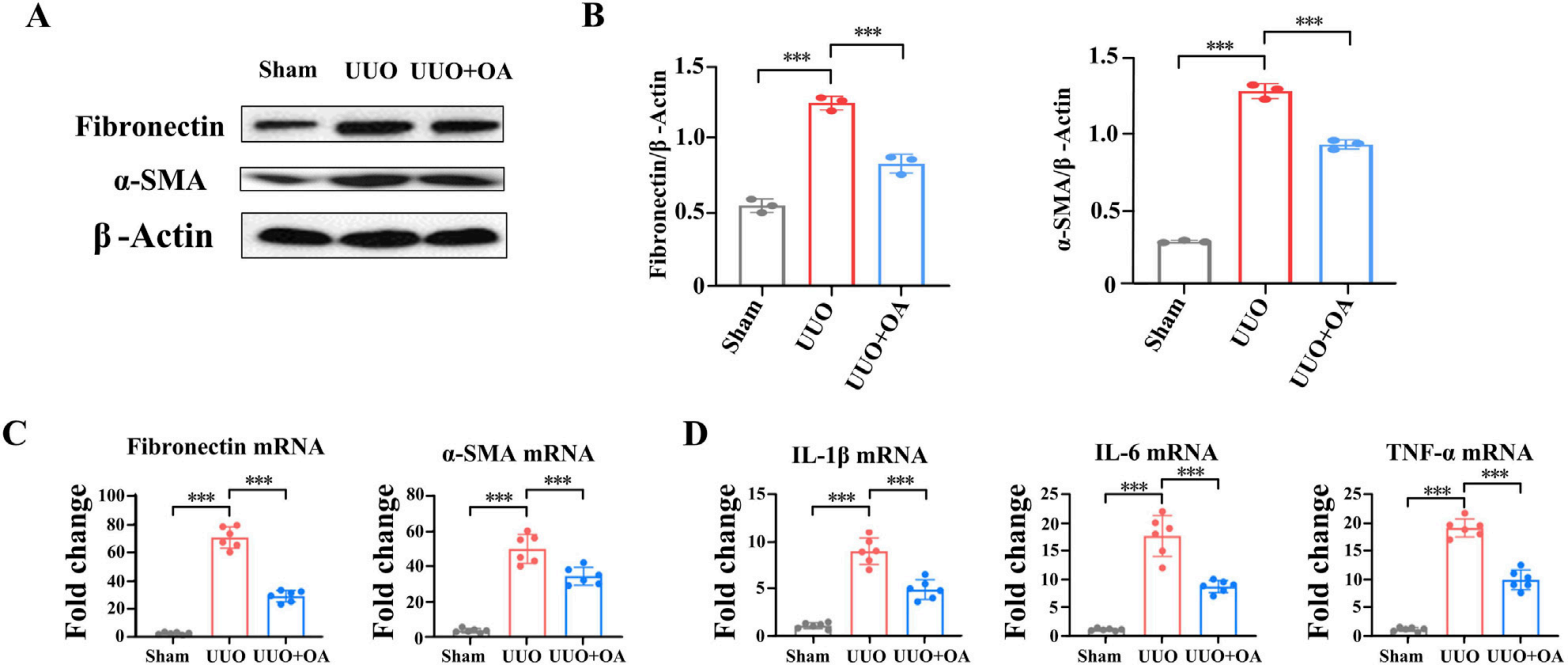
Oroxylin A reduces renal fibrosis and inflammation in UUO mice. **(A, B)** Western blot analysis for fibronectin and α-SMA protein levels in UUO mice (n = 3). **(C)** qRT-PCR analysis of fibronectin and α-SMA mRNA expression in UUO mice (n = 6). **(D)** qRT-PCR analysis of Il-1β, Il-6, and TNF-α mRNA expression in UUO mice (n = 6). Data are represented as the mean ± sd. ***p < 0.001 and **p < 0.01.

We analyzed the expression levels of proteins through immunostaining and Western blotting. Compared to the sham surgery group, the expression levels of fibronectin and α-SMA in the renal tissue of UUO mice were significantly increased. However, OA treatment remarkably restrained the accumulation of fibronectin and α-SMA (Figures 5A–D). Western blot and RT-PCR analyses showed that OA reduced the expression of fibrosis markers in obstructed kidneys (Figures 6A–C). Collectively, these results further confirmed that OA treatment reduced the expression levels of fibronectin and α-SMA in UUO mice.

### 3.4 OA restrained kidney inflammation in UUO mice

The process of renal fibrosis is accompanied by a large number of inflammatory reactions. According to reports, OA has anti-inflammatory effects (Liu et al., 2024). We then evaluated the influence of OA treatment on various inflammatory factors in renal tissue. The mRNA levels of IL-1β, IL-6, and TNF-α were significantly increased in the obstructed kidneys of UUO mice. However, OA treatment remarkably decreased the production of the abovementioned inflammatory factors (Figure 6D). These results further confirmed that OA treatment might suppress the inflammatory response in UUO mice.

### 3.5 OA inhibited renal fibrosis progression by activating Sirt1

**FIGURE 7:**
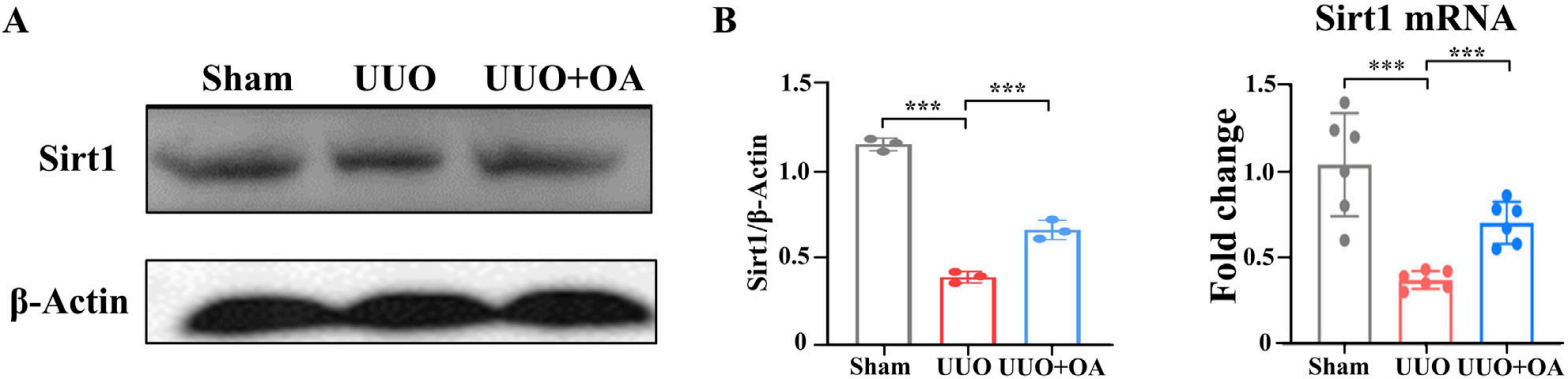
Oroxylin A treatment exerted protection via Sirt1. **(A)** Western blot analysis for Sirt1 protein levels of kidney tissue in UUO mice at 14 days after surgery. **(B)** qRT-PCR analysis of Sirt1 mRNA expression in the obstructed mouse kidney at 14 days after UUO surgery (n = 6). Data are represented as the mean ± sd. ***p < 0.001 and **p < 0.01.

Previous studies have confirmed that OA protects mice from dox-induced acute cardiac injury by activating the Sirt1 signaling pathway (Zhang et al., 2021; Yao M. et al., 2022). We aimed to determine whether OA activated the Sirt1 signaling pathway. Therefore, we detected the expression level of Sirt1 in the kidney tissue of UUO mice. As expected, the expression level of Sirt1 in the kidneys of UUO mice was significantly reduced (Figures 7A, B), whereas these reductions were largely reversed by OA administration. The above data suggested that OA could activate the Sirt1 signaling pathway, which might be an essential reason for the anti-fibrotic effect of OA.

### 3.6 OA reversed TGF-β1-induced pro-fibrotic markers by activating the Sirt1 signaling pathway

**FIGURE 8:**
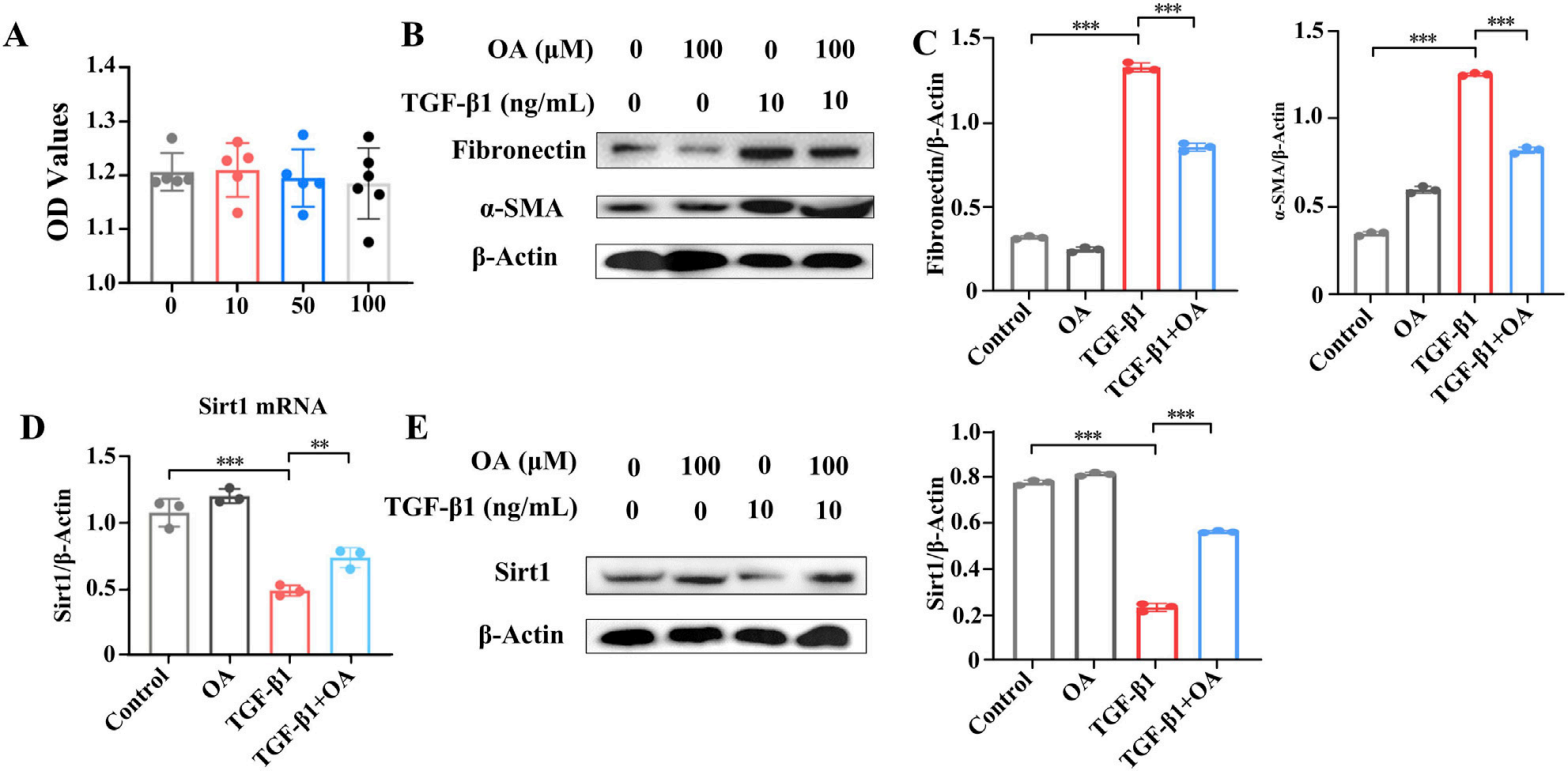
Oroxylin A inhibited TGF-β1-induced pro-fibrotic markers of HK-2 cells through enhanced Sirt1 expression. **(A)** Cell viability analysis of HK-2 cells pretreated with different concentrations of oroxylin A for 24 h (n = 6). **(B, C)** Western blot analysis of fibronectin and α-SMA protein levels in HK-2 cells pretreated with oroxylin A (10 μM), followed by incubation with 10 ng/mL TGF-β1 for 24 h (n = 3). **(D)** qRT-PCR analysis and **(E)** Western blot analysis of Sirt1 in HK-2 cells pretreated with oroxylin A, followed by incubation with 10 ng/mL TGF-β1 for 24 h (n = 3). Data are represented as the mean ± sd. ***p < 0.001 and **p < 0.01.

We then investigated the protective effects of OA on HK-2 cells treated with TGF-β1. First, we assessed the cytotoxicity of OA in HK-2 cells. OA showed no obvious cytotoxicity toward HK-2 cells at different concentrations (10, 50, and 100 μM), as shown in Figure 8A. Therefore, a concentration of 10 μM of OA was used in subsequent experiments. In cultured HK-2 human kidney epithelial cells, TGF-β1 significantly increased the expression levels of fibronectin and α-SMA. *In vitro*, OA pretreatment significantly reduced the expression of fibronectin and α-SMA (Figures 8B, C). We then wondered whether OA plays a role in renal fibrosis by regulating Sirt1 levels in renal tubular epithelial cells. *In vitro*, Sirt1 levels were significantly reduced, but they were upregulated after OA treatment (Figures 8D, E). The results further confirmed that OA also has a strong anti-fibrotic effect *in vitro* by increasing the expression of Sirt1.

### 3.7 OA inhibited fibrosis by activating Sirt1 and inhibiting the TGF-β/Smad3 pathway

**FIGURE 9:**
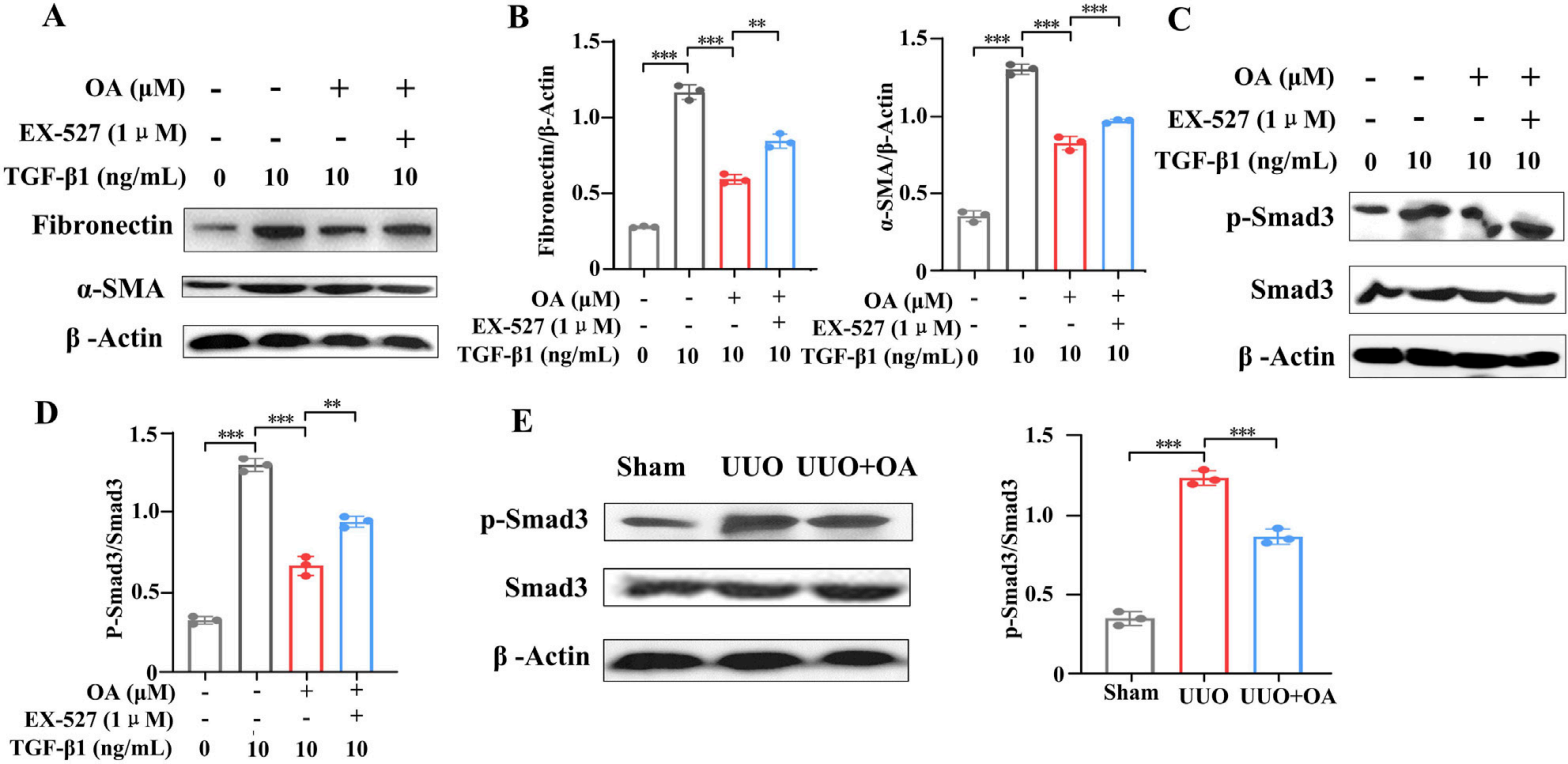
Sirt1 depletion abolished the protection of oroxylin A. HK-2 cells pretreated with EX-527, oroxylin A (10 μM), followed by incubation with 10 ng/mL TGF-β1 for 24 h. **(A, B)** Western blot analysis of fibronectin and α-SMA protein expression in HK-2 cells (n = 3). **(C, D)** Western blot analysis of p-Smad3 and Smad3 protein levels in HK-2 cells (n = 3). **(E)** Western blot analysis for p-Smad3 and Smad3 protein levels in UUO mice at 14 days after surgery (n = 3). Data are represented as the mean ± sd. ***p < 0.001 and **p < 0.01.

The cells were pretreated with a specific Sirt1 inhibitor (EX-527) following treatment with TGF-β1. The results showed that OA impeded TGF-β1-stimulated aberrant expression of fibronectin and α-SMA, whereas EX-527 pretreatment eliminated the protective effect of OA (Figures 9A, B). Together, the above results support that OA exerts an anti-fibrotic effect partly through the promotion of the activity of Sirt1. Given the important role of TGF-β/Smad3 signaling in renal fibrosis, the expression of Smad3 was examined. The results showed that OA inhibited TGF-β-induced Smad3 activation, and EX-527 pretreatment abrogated the suppressive effect of OA on Smad3 activation (Figures 9C, D). Following the *in vitro* results, OA treatment markedly inhibited the activation of Smad3 in UUO mice, thereby ameliorating renal fibrosis (Figure 9E). Collectively, these results indicated that OA repressed renal fibrosis by activating the expression of Sirt1 and blocking the TGF-β/Smad3 pathway.

### 3.8 OA mediated the TGF-β/Smad3 signaling pathway by activating Sirt1

**FIGURE 10:**
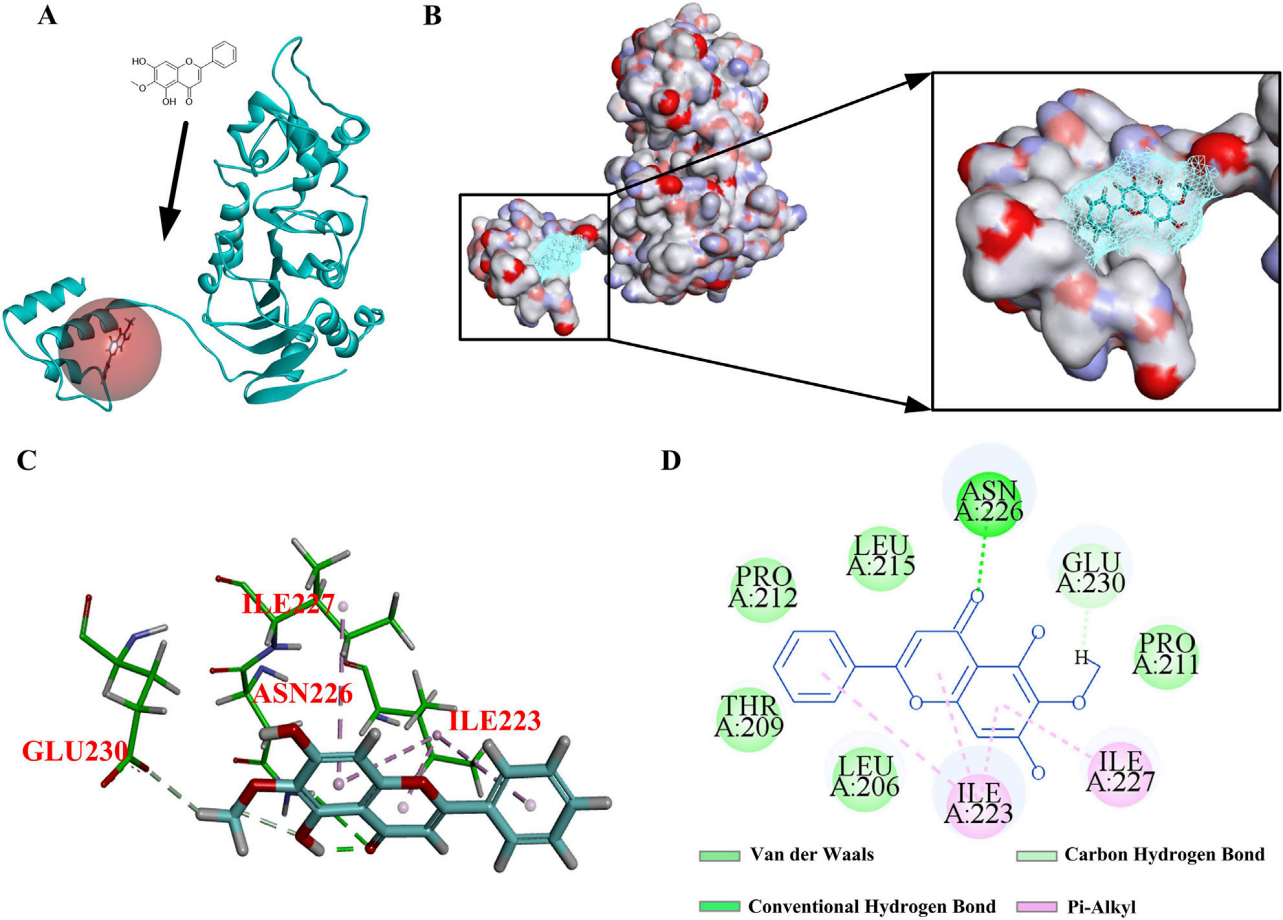
Interaction diagram under OA and Sirt1. **(A, B)** 3D diagrams of the interaction between OA and Sirt1. **(C, D)** 2D diagrams of the interaction between OA and Sirt1.

In order to better analyze the rationality of the new molecular structure, molecular docking was selected to observe the interaction between the active site of the receptor and the compound. In general, interactions, including hydrogen bonding and hydrophobic and electrostatic interactions, play an important role in the stability of these receptor–ligand complexes in docking analysis (Jia et al., 2018). Based on the above research, we hypothesized that OA might regulate downstream signaling pathways by activating Sirt1, alleviate the degree of renal fibrosis, and have a protective effect on the kidneys. According to research reports, GLU230 was a key amino acid in the Sirt1 pocket (Dai et al., 2015). Resveratrol is a typical Sirt1 agonist that can target Sirt1 to form hydrogen bonds with the key amino acid GLU230. Next, molecular docking was used to investigate the binding of OA and Sirt1. The results showed that OA binds to the Sirt1 active site (Figures 10A, B). OA formed hydrogen bonds with the key amino acid GLU230, and it also formed hydrogen bonds with the amino acid ASN226 that resveratrol did not have, which might enhance the binding ability of OA to Sirt1. In addition, OA could also form pi–alkyl interactions, which are a type of hydrophobic interactions with ILE223 and ILE227 in the active site (Figures 10C, D). Altogether, this might be the main reason why OA is more stable than resveratrol binding.

## 4 Discussion

Currently, the mechanism of renal fibrosis is still largely unknown, and there is no effective treatment. The major findings of this study showed that OA ameliorates renal fibrosis by activating the expression and activity of Sirt1 and inhibiting the TGF-β/Smad3 signaling pathway. Thus, our current findings provide a new pathway and promising treatment for renal fibrosis and CKD.

OA is a natural compound and has a wide range of pharmacological effects (Yao J.Y. et al., 2022). Previous studies mainly focused on the antitumor effects of OA (Yu et al., 2023; Zhu Q. Q. et al., 2020), revealing that OA has a very strong inhibitory effect on tumor invasion and metastasis. As reported, OA can exert an anti-colon cancer effect by promoting the apoptosis of colon cancer cells (Qiao et al., 2015). Furthermore, OA can also prevent the invasion of non-small-cell lung cancer cells (Huo et al., 2022; Wei et al., 2015). An increasing number of studies have confirmed that OA plays a significant role in liver fibrosis, acute liver injury, osteoarthritis, and myocardial cell injury (Jin et al., 2018; Chen et al., 2021). Our study reports the role of OA in the treatment of renal fibrosis for the first time. We fully confirmed that OA had a significant anti-fibrotic effect. Subsequent results confirmed that OA inhibited renal fibrosis by regulating Sirt1 activity. Sirt1 is one of the most interesting members, with multiple functions, including cellular metabolism, immune response, and senescence (Huang et al., 2021; Shi et al., 2024; Cui et al., 2022; Kadono et al., 2022). Sirt1 has protective effects on various kidney diseases, such as acute renal injury, diabetes nephropathy, and CAD (Shu et al., 2024; Zhong and Zhang, 2024; Yan et al., 2022). Sirt1 ameliorated renal fibrosis by reducing endoplasmic reticulum stress (Chang et al., 2016). In our study, OA treatment remarkably mitigated renal fibrosis and conserved kidney function by activating Sirt1 and suppressing the TGF-β/Smad3 pathway. The above results support that OA exerts an anti-fibrotic effect at least partly through promoting the activity of Sirt1.

Some effective drugs for the prevention or treatment of renal fibrosis have also been found in abundant natural resources. For example, quercetin alleviates renal fibrosis by inhibiting the TGF-β/Smad3 signaling pathway. However, *in vitro*, it is administered orally twice as much as OA (Lu et al., 2018). Epigallocatechin gallate inhibits the TGF-β/Smad3 signaling pathway and attenuates renal fibrosis (Zhu Y. et al., 2020). Pectin alleviates renal fibrosis by inhibiting Smad3 and TGF-β/Smad3 signaling (Ren et al., 2022). The TGF-β/Smad3 signaling pathway is one of the classic signaling pathways for the treatment of renal fibrosis. OA inhibits the TGF-β/Smad3 signaling pathway through different targets and has great potential for the treatment of renal fibrosis.

In our study, through the UUO mouse model, we observed that OA administration improved renal fibrosis and renal function in mice. It upregulated the expression of Sirt1 and inhibited the TGF-β/Smad3 pathway, thus significantly and markedly improving renal fibrosis and renal function. OA mainly formed hydrogen bonds with the key amino acid GLU230 in the active site. Among them, hydrogen bond interactions are more critical for evaluating the binding affinity and stable conformations. The results showed that OA had good binding ability to proteins. The above experimental results together proved that OA exerts an anti-fibrosis effect through the activation of Sirt1 activity. Furthermore, based on previous studies, we speculated that OA had potential and great benefits for the treatment of human diseases in clinical settings. First, OA is the main effective component of astragalus, which is the active ingredient of natural food sources. Second, OA has multiple biological effects and is safe without side effects and cytotoxicity. Third, together with previous reports, it collectively reveals that OA exerts an anti-fibrotic effect. In conclusion, our findings present strong evidence for OA in the clinical therapy of renal fibrosis. Next, we will explore the relationship between OA and Sirt1 activity in more depth.

## 5 Conclusion

We studied the druggability and anti-renal fibrosis effect of OA based on gene and protein levels. The results showed that physicochemical and ADMET properties of OA were within an acceptable range, and OA could significantly reduce the expression of fibrosis markers. The renal fibrosis-related mRNA expression and proteins levels of fibronectin and α-SMA were significantly reduced compared to the model group. Inflammation is a hallmark of renal fibrosis. Through the treatment of OA, we found that the mRNA expression levels of inflammatory factors (IL-1β, IL-6, and TNF-α) decreased, while the mRNA expression level of Sirt1 increased. OA could promote the expression of Sirt1. Through protein and gene validation, we found that the anti-fibrosis renal protective effect of OA was tightly linked to Sirt1 and the TGF-β/Smad3 signaling pathway. OA might serve as a treatment for CKD, considering its anti-fibrosis effect. These claims need further investigation.

## Data Availability

The original contributions presented in the study are included in the article/Supplementary Material; further inquiries can be directed to the corresponding authors.

## References

[B1] Bai D. Sun T. Zhao J. Du J. Bu X. Cao W. (2021). Oroxylin A maintains the colonic mucus barrier to reduce disease susceptibility by reconstituting a dietary fiber-deprived gut microbiota. Cancer Lett. 515, 73–85. 10.1016/j.canlet.2021.05.018 34052330

[B2] Ceccotti E. Chiabotto G. Cedrino M. Gambella A. Delsedime L. Ghigo A. (2024). Extracellular vesicles derived from human liver stem cells counteract chronic kidney disease development and cardiac dysfunction in remnant kidney murine model: the possible involvement of proteases. Biomedicines 12 (7), 1517. 10.3390/biomedicines12071517 39062090 PMC11274379

[B3] Chang J. W. Kim H. Baek C. H. Lee R. B. Yang W. S. Lee S. K. (2016). Up-Regulation of Sirt1 reduces endoplasmic reticulum stress and renal fibrosis. Nephron 133 (2), 116–128. 10.1159/000447067 27255945

[B4] Chen D. H. Zheng G. Zhong X. Y. Lin Z. H. Yang S. W. Liu H. X. (2021). Oroxylin A attenuates osteoarthritis progression by dual inhibition of cell inflammation and hypertrophy. Food Funct. 12 (1), 328–339. 10.1039/d0fo02159h 33300913

[B5] Chen W. Zhang Z. Yao Z. Wang L. Zhang F. Shao J. (2018). Activation of autophagy is required for Oroxylin A to alleviate carbon tetrachloride-induced liver fibrosis and hepatic stellate cell activation. Int. Immunopharmacol. 56, 148–155. 10.1016/j.intimp.2018.01.029 29414645

[B6] Chen Y. Zheng J. Mo L. Chen F. Li R. Wang Y. (2024). Oroxylin A suppresses breast cancer-induced osteoclastogenesis and osteolysis as a natural RON inhibitor. Phytomedicine 129, 155688. 10.1016/j.phymed.2024.155688 38728920

[B7] Cui Z. Zhao X. Amevor F. K. Du X. Wang Y. Li D. (2022). Therapeutic application of quercetin in aging-related diseases: Sirt1 as a potential mechanism. Front. Immunol. 13, 943321. 10.3389/fimmu.2022.943321 35935939 PMC9355713

[B8] Dai H. Case A. W. Riera T. V. Considine T. Lee J. E. Hamuro Y. (2015). Crystallographic structure of a small molecule Sirt1 activator-enzyme complex. Nat. Commun. 6, 7645. 10.1038/ncomms8645 26134520 PMC4506539

[B9] Daina A. Michielin O. Zoete V. (2017). SwissADME: a free web tool to evaluate pharmacokinetics, drug-likeness and medicinal chemistry friendliness of small molecules. Sci. Rep. 7, 42717. 10.1038/srep42717 28256516 PMC5335600

[B10] Huang Q. Su H. Qi B. Wang Y. Yan K. Wang X. (2021). A Sirt1 activator, ginsenoside rc, promotes energy metabolism in cardiomyocytes and neurons. J. Am. Chem. Soc. 143 (3), 1416–1427. 10.1021/jacs.0c10836 33439015

[B11] Huo T. X. Wang X. P. Yu Z. Kong B. He Y. Guo Q. L. (2022). Oroxylin A inhibits the migration of hepatocellular carcinoma cells by inducing NAG-1 expression. Acta Pharmacol. Sin. 43 (3), 724–734. 10.1038/s41401-021-00695-4 34117368 PMC8888648

[B12] Jia W. Q. Jing Z. Liu X. Feng X. Y. Liu Y. Y. Wang S. Q. (2018). Virtual identification of novel PPARα/γ dual agonists by scaffold hopping of saroglitazar. J. Biomol. Struct. Dyn. 36 (13), 3496–3512. 10.1080/07391102.2017.1392363 29081262

[B13] Jin H. Lian N. Bian M. Zhang C. Chen X. Shao J. (2018). Oroxylin A prevents alcohol-induced hepatic steatosis through inhibition of hypoxia inducible factor 1alpha. Chem. Biol. Interact. 285, 14–20. 10.1016/j.cbi.2018.02.025 29476730

[B14] Ju Y. Li Z. Deng Y. Tong A. Zhou L. Lu Y. (2016). Identification of novel Bace1 inhibitors by combination of pharmacophore modeling, structure-based design and *in vitro* assay. Curr. Comput. Aided Drug Des. 12 (1), 73–82. 10.2174/1573409912666160222113103 26899408

[B15] Kadono K. Kageyama S. Nakamura K. Hirao H. Ito T. Kojima H. (2022). Myeloid ikaros-Sirt1 signaling axis regulates hepatic inflammation and pyroptosis in ischemia-stressed mouse and human liver. J. Hepatol. 76 (4), 896–909. 10.1016/j.jhep.2021.11.026 34871625 PMC9704689

[B16] Kim D. H. Jeon S. J. Son K. H. Jung J. W. Lee S. Yoon B. H. (2007). The ameliorating effect of Oroxylin A on scopolamine-induced memory impairment in mice. Neurobiol. Learn Mem. 87 (4), 536–546. 10.1016/j.nlm.2006.11.005 17196405

[B17] Ledo G. V. A. Lozada-Perezmitre E. Pruinelli L. Landeros-Olvera E. Gómez F. M. I. (2024). Mobile applications in patients with chronic kidney disease: a systematic review. Stud. Health Technol. Inf. 315, 380–385. 10.3233/SHTI240174 39049287

[B18] Li P. Liu Y. Qin X. Chen K. Wang R. Yuan L. (2021). Sirt1 attenuates renal fibrosis by repressing HIF-2α. Cell Death Discov. 7 (1), 59. 10.1038/s41420-021-00443-x 33758176 PMC7987992

[B19] Liu Q. Zhang Y. Yang S. Wu Y. Wang J. Yu W. (2017). PU.1-deficient mice are resistant to thioacetamide-induced hepatic fibrosis: PU.1 finely regulates Sirt1 expression via transcriptional promotion of *miR-34a* and *miR-29c* in hepatic stellate cells. Biosci. Rep. 37 (6), BSR20170926. 10.1042/BSR20170926 29162670 PMC5725609

[B20] Liu T. Zhu S. Yang Y. Qin W. Wang Z. Zhao Z. (2024). Oroxylin A ameliorates ultraviolet radiation-induced premature skin aging by regulating oxidative stress via the Sirt1 pathway. Biomed. Pharmacother. 171, 116110. 10.1016/j.biopha.2023.116110 38198955

[B21] Liu Y. Wang X. Li W. Xu Y. Zhuo Y. Li M. (2020). Oroxylin A reverses hypoxia-induced cisplatin resistance through inhibiting HIF-1α mediated XPC transcription. Oncogene 39 (45), 6893–6905. 10.1038/s41388-020-01474-x 32978517

[B22] Lu H. Wu L. Liu L. Ruan Q. Zhang X. Hong W. (2018). Quercetin ameliorates kidney injury and fibrosis by modulating M1/M2 macrophage polarization. Biochem. Pharmacol. 154, 203–212. 10.1016/j.bcp.2018.05.007 29753749

[B23] Miguel V. Tituaña J. Herrero J. I. Herrero L. Serra D. Cuevas P. (2021). Renal tubule cpt1a overexpression protects from kidney fibrosis by restoring mitochondrial homeostasis. J. Clin. Invest. 131 (5), e140695. 10.1172/JCI140695 33465052 PMC7919728

[B24] Oyama N. Kawaguchi M. Itaka K. Kawakami S. (2021). Efficient messenger RNA delivery to the kidney using renal pelvis injection in mice. Pharmaceutics 13 (11), 1810. 10.3390/pharmaceutics13111810 34834225 PMC8619888

[B25] Qiao C. Wei L. Dai Q. Zhou Y. Yin Q. Li Z. (2015). UCP2-related mitochondrial pathway participates in Oroxylin A-induced apoptosis in human colon cancer cells. J. Cell Physiol. 230 (5), 1054–1063. 10.1002/jcp.24833 25251374

[B26] Ren Q. Wang B. Guo F. Huang R. Tan Z. Ma L. (2022). Natural flavonoid pectolinarigenin alleviated hyperuricemic nephropathy via suppressing tgfβ/SMAD3 and JAK2/STAT3 signaling pathways. Front. Pharmacol. 12, 792139. 10.3389/fphar.2021.792139 35153751 PMC8829971

[B27] Ruiz-Ortega M. Rayego-Mateos S. Lamas S. Ortiz A. Rodrigues-Diez R. R. (2020). Targeting the progression of chronic kidney disease. Nat. Rev. Nephrol. 16 (5), 269–288. 10.1038/s41581-019-0248-y 32060481

[B28] Shen M. Guo M. Wang Z. Li Y. Kong D. Shao J. (2020). ROS-dependent inhibition of the PI3K/Akt/mTOR signaling is required for Oroxylin A to exert anti-inflammatory activity in liver fibrosis. Int. Immunopharmacol. 85, 106637. 10.1016/j.intimp.2020.106637 32512269

[B29] Shi H. Xie X. Zheng S. Chen H. Liu C. Li S. (2024). Endotoxin tolerance ameliorates lipopolysaccharide/D-galactosamine-induced acute liver failure by negative regulation of the NF-κB/NLRP3 and activation of Nrf2/HO-1 via Sitr1. Int. Immunopharmacol. 132, 111994. 10.1016/j.intimp.2024.111994 38581992

[B30] Shu G. Wang C. Song A. Zheng Z. Zheng S. Song Y. (2024). Water extract of earthworms mitigates kidney injury triggered by oxidative stress via activating intrarenal Sirt1/Nrf2 cascade and ameliorating mitochondrial damage. J. Ethnopharmacol. 335, 118648. 10.1016/j.jep.2024.118648 39089659

[B31] Speer T. Dimmeler S. Schunk S. J. Fliser D. Ridker P. M. (2022). Targeting innate immunity-driven inflammation in CKD and cardiovascular disease. Nat. Rev. Nephrol. 18 (12), 762–778. 10.1038/s41581-022-00621-9 36064794

[B32] Sureshbabu A. Muhsin S. A. Choi M. E. (2016). TGF-β signaling in the kidney: profibrotic and protective effects. Am. J. Physiol. Ren. Physiol. 310 (7), F596–F606. 10.1152/ajprenal.00365.2015 PMC4824143 26739888

[B33] Wang J. Zhou F. Xiong C. E. Wang G. P. Chen L. W. Zhang Y. T. (2023). Serum sirtuin1: a potential blood biomarker for early diagnosis of Alzheimer's disease. Aging (Albany NY) 15 (18), 9464–9478. 10.18632/aging.205015 37742223 PMC10564418

[B34] Wang P. X. Mu X. N. Huang S. H. Hu K. Sun Z. G. (2024). Cellular and molecular mechanisms of Oroxylin A in cancer therapy: recent advances. Eur. J. Pharmacol. 969, 176452. 10.1016/j.ejphar.2024.176452 38417609

[B35] Wei L. Zhou Y. Qiao C. Ni T. Li Z. You Q. (2015). Oroxylin A inhibits glycolysis-dependent proliferation of human breast cancer via promoting Sirt3-mediated SOD2 transcription and HIF1α destabilization. Cell Death Dis. 6 (4), e1714. 10.1038/cddis.2015.86 25855962 PMC4650553

[B36] Yan J. Wang J. He J. C. Zhong Y. (2022). Sirtuin 1 in chronic kidney disease and therapeutic potential of targeting Sirtuin 1. Front. Endocrinol. (Lausanne) 13, 917773. 10.3389/fendo.2022.917773 35795148 PMC9251114

[B37] Yang S. Cheng Y. Wang X. Yue S. Wang X. Tang L. (2024). Chinese herbal decoction, Yi-Qi-Jian-Pi formula exerts anti-hepatic fibrosis effects in mouse models of CCl_4_-induced liver fibrosis. Heliyon 10 (5), e26129. 10.1016/j.heliyon.2024.e26129 38434258 PMC10907526

[B38] Yao J. Y. J. Y. Xu S. Sun Y. N. Xu Y. Guo Q. L. Wei L. B. (2022). Novel CDK9 inhibitor Oroxylin A promotes wild-type P53 stability and prevents hepatocellular carcinoma progression by disrupting both MDM2 and Sirt1 signaling. Acta Pharmacol. Sin. 43 (4), 1033–1045. 10.1038/s41401-021-00708-2 34188177 PMC8975870

[B39] Yao M. M. Qin S. Xiong J. Xin W. Guan X. Gong S. (2022). Oroxylin A ameliorates AKI-to-CKD transition through maintaining PPARα-BNIP3 signaling-mediated mitochondrial homeostasis. Front. Pharmacol. 13, 935937. 10.3389/fphar.2022.935937 36081929 PMC9445212

[B40] Yu P. Li J. Luo Y. Sun J. Hu Y. Lin B. (2023). Mechanistic role of scutellaria baicalensis georgi in breast cancer therapy. Am. J. Chin. Med. 51 (2), 279–308. 10.1142/S0192415X23500155 36655686

[B41] Yu X. Y. Sun Q. Zhang Y. M. Zou L. Zhao Y. Y. (2022). TGF-β/Smad signaling pathway in tubulointerstitial fibrosis. Front. Pharmacol. 13, 860588. 10.3389/fphar.2022.860588 35401211 PMC8987592

[B42] Zhang C. Bian M. Chen X. Jin H. Zhao S. Yang X. (2018). Oroxylin A prevents angiogenesis of LSECs in liver fibrosis via inhibition of YAP/HIF-1α signaling. J. Cell Biochem. 119 (2), 2258–2268. 10.1002/jcb.26388 28857294

[B43] Zhang W. B. Zheng Y. F. Wu Y. G. (2021). Protective effects of Oroxylin A against doxorubicin-induced cardiotoxicity via the activation of Sirt1 in Mice. Oxid. Med. Cell Longev. 2021, 6610543. 10.1155/2021/6610543 33542782 PMC7840263

[B44] Zhong W. Jiang Y. Wang H. Luo X. Zeng T. Huang H. (2024). Fibroblast growth factor 21 alleviates unilateral ureteral obstruction-induced renal fibrosis by inhibiting Wnt/β-catenin signaling pathway. Biochim. Biophys. Acta Mol. Cell Res. 1871 (2), 119620. 10.1016/j.bbamcr.2023.119620 37926157

[B45] Zhong X. Zhang J. (2024). RUNX3-activated apelin signaling inhibits cell proliferation and fibrosis in diabetic nephropathy by regulation of the Sirt1/Foxo pathway. Diabetol. Metab. Syndr. 16 (1), 167. 10.1186/s13098-024-01393-x 39014438 PMC11253400

[B46] Zhu J. Li K. Xu L. Cai Y. Chen Y. Zhao X. (2021). Discovery of novel selective PI3Kγ inhibitors through combining machine learning-based virtual screening with multiple protein structures and bio-evaluation. J. Adv. Res. 36, 1–13. 10.1016/j.jare.2021.04.007 35127160 PMC8800018

[B47] Zhu Q. Q. Yang X. Y. Zhang X. J. Yu C. J. Pang Q. Q. Huang Y. W. (2020). EGCG targeting Notch to attenuate renal fibrosis *via* inhibition of TGFβ/Smad3 signaling pathway activation in streptozotocin-induced diabetic mice. Food Funct. 11 (11), 9686–9695. 10.1039/d0fo01542c 33057539

[B48] Zhu Y. Guo Y. Liu M. Wei L. Wang X. (2020). An Oroxylin A-loaded aggregation-induced emission active polymeric system greatly increased the antitumor efficacy against squamous cell carcinoma. J. Mater Chem. B 8 (10), 2040–2047. 10.1039/c9tb01818b 32100790

