# Corrigendum: Efficacy of Oroxylin A in ameliorating renal fibrosis with emphasis on Sirt1 activation and TGF-β/Smad3 pathway modulation

**DOI:** 10.3389/fphar.2024.1548193

**Published:** 2025-01-06

**Authors:** Guangzhuang Li, Sentao Xian, Xianchao Cheng, Yunhua Hou, Wenqing Jia, Yukui Ma

**Affiliations:** ^1^ School of Bioengineering, Qilu University of Technology (Shandong Academy of Sciences), Jinan, Shandong, China; ^2^ School of Chemistry and Chemical Engineering, Qilu Normal University, Jinan, China; ^3^ Tianjin Key Laboratory on Technologies Enabling Development of Clinical Therapeutics and Diagnostics (Theranostics), School of Pharmacy, Tianjin Medical University, Tianjin, China

**Keywords:** Oroxylin A, chronic kidney disease, renal fibrosis, Sirt1, molecular docking

In the published article, there was an error in **Affiliation 1**. Instead of “School of Bioengineering, Shandong Academy of Sciences, Qilu University of Technology, Jinan, China,” it should be “School of Bioengineering, Qilu University of Technology (Shandong Academy of Sciences), Jinan, Shandong, China.”

The authors apologize for this error and state that this does not change the scientific conclusions of the article in any way. The original article has been updated.

